# Physical activity in adolescents of different family socioeconomic status: the moderating role of gender

**DOI:** 10.3389/fped.2025.1559554

**Published:** 2025-03-20

**Authors:** Ting Zhang, Ming Li, Feng Zhang, Xiaofang Yang, Pengwei Sun, Xiaojian Yin, Yuan Liu, Yaru Guo

**Affiliations:** ^1^ School of Physical Education, Suzhou University, Suzhou, China; ^2^ School of Physical Education, Xizang Minzu University, Xianyang, China; ^3^ College of Physical Education and Health, East China Normal University, Shanghai, China; ^4^ Key Laboratory of Adolescent Health Assessment and Exercise Intervention of Ministry of Education, East China Normal University, Shanghai, China; ^5^ College of Physical Education, Jiangxi Normal University, Nanchang, China; ^6^ College of Economics and Management, Shanghai Institute of Technology, Shanghai, China; ^7^ School of Physical Education, Shanghai University, Shanghai, China

**Keywords:** adolescent, physical activity, family socioeconomic status, gender, MVPA

## Abstract

**Background:**

To investigate the association between family socioeconomic status (SES) and the physical activity (PA) levels of adolescents, as well as the moderating effect of gender on this relationship.

**Methods:**

A total of 10,327 Chinese adolescents aged 12–17 were recruited to complete questionnaires regarding their SES and PA levels. “Physical Activity Questionnaire for Children and Adolescents Aged 7–18 Years” was utilized to examine the specific items, intensity, duration, and frequency of PA. The Kruskal–Wallis test and the Mann–Whitney U test were employed to compare PA time among adolescents across different groups. To analyze the interaction effect between gender and SES, the Scheirer-Ray-Hare test was conducted using R software. Additionally, SPSSAU was utilized to examine the moderating effect of gender and SES on PA.

**Result:**

(1) Different intensities of PA: Overall, the times for LPA, MPA, VPA, MVPA, and TPA in the low SES group were 190 (100, 400) minutes, 190 (80, 400) minutes, 60 (0, 160) minutes, 290 (150, 570) minutes, and 580 (345, 925) minutes, respectively.The duration of PA at each intensity level in the low SES group was significantly lower compared to that in the middle and high SES groups (all *P*-values < 0.05). (2) Different PA types: The low SES group exhibited the shortest durations for both transportation PA (260 min) and exercise PA (155 min), while household PA was the longest (15 min)(all *P*-values < 0.05). (3) Moderating effect test: After controlling for relevant variables, the interaction term coefficient between gender and SES was statistically significant (B = −19.141, *t* = −2.059, *P* *<* 0.05).

**Conclusion:**

Adolescents from low SES backgrounds exhibited the lowest levels of MVPA and TPA, which were mainly manifested in transportation PA and exercise PA. Gender moderates the relationship between SES and MVPA, with different SES levels having a more pronounced effect on PA in boys than in girls.

## 1 Introduction

Physical Activity (PA) is one of the key health behaviors to reduce the risk of morbidity and mortality (1, 2). Maintaining an adequate level of PA is essential for promoting physical and mental healthy for adolescents (3). In response to the global decline in PA levels, the World Health Organization (WHO) and various countries have issued PA guidelines recommending that children and adolescents engage in at least 60 min of moderate to vigorous physical activity (MVPA) daily (4, 5, 6). Despite global efforts to promote PA, over 80% of adolescents fail to meet this recommendation. This phenomenon is particularly pronounced among girls and lower socioeconomic status (SES) (7). The COVID-19 pandemic has further exacerbated this issue, leading to a significant reduction in PA across all age groups (8–11). Given these concerns, it is essential to explore how SES influences PA participation.

The relationship between SES and PA is complex and multifaceted, with significant disparities observed across different socioeconomic groups (12). While it is commonly believed that higher SES individuals are generally believed to engage in more PA than those with lower SES (13). Some studies have found no significant differences in PA levels across SES groups, and in certain cases, lower SES groups exhibit higher levels of PA in transportation PA (12, 14). This discrepancy may be attributed to the different types of PA engaged in by various SES groups. Cultural and social norms within different SES groups may influence attitudes toward PA. Higher SES groups may place a greater emphasis on PA as a means of maintaining health and social status, while lower SES groups may prioritize PA for practical purposes, such as commuting or work-related activities (14). Variations in access to recreational and fitness facilities due to differing SES, in conjunction with temporal limitations imposed by occupational and familial obligations, may significantly influence an individual's engagement in PA (15). These differences in PA types and contexts suggest that SES influences not only the quantity but also the nature of PA, which may have implications for health outcomes. Therefore, distinguishing the PA types is critical to addressing the research gap.

At the same time, the PA levels of boys and girls differ to some extent, with boys generally being more active than girls. However, gender-based differences in PA are also observed across various SES groups. Prior studies have documented that male adolescents in high-income countries exhibit the lowest prevalence of physical inactivity, whereas their counterparts in low-income countries display the highest prevalence. However, this pattern is not evident among girls (9). Additionally, the calculation methods for the SES index vary across different studies, which can introduce bias and complicate comparisons between research findings. Some studies use income or education level as proxies for SES, while others employ composite indices that include occupation, neighborhood quality, and access to resources (12, 13, 16). These methodological differences highlight the need for a standardized approach to measuring SES in PA research, particularly when examining its interaction with gender. Therefore, this study employs the SES calculation method utilized in the 2015 Programme for International Student Assessment (PISA) (17) to determine SES levels. This methodology incorporates parental educational attainment, occupational status, and household income into a composite index, thereby providing a more objective and comprehensive representation of the SES of adolescents. Based on the aforementioned empirical evidence, we postulate the following hypothesis: SES is negatively associated with PA levels among adolescents, with gender potentially serving as a moderating variable in this relationship.

This study employs a scientifically rigorous methodology to quantify SES levels, to understand the characteristics of PA among adolescents of different SES, and the role of gender within it. This study aims to provide a theoretical foundation for developing equitable intervention strategies to mitigate physical inactivity among adolescents across varying SES, thereby contributing to the enhancement of overall PA levels among adolescents in China.

## 2 Participants and method

### 2.1 Participants

#### 2.1.1 Calculation of sample size

The sample size for each stratum was determined utilizing the formula for sample size calculation: *N* = *deff u*^2^*p(*1-p*)*/*δ*^2^*.* The stratified sampling design effect (deff) was 0.8, with *u* = 1.96 at *α* = 0.05. Based on previous studies (18, 19, 20), the estimated detection rate of adolescent psychological problems was 20%. Tolerance error (*δ*) = 0.10p. It is estimated that a sample size of 1,229 individuals is required for each age cohort. A total of 7,374 samples were required for the six age groups. Considering a 10% non-response rate, the test sample size was calculated as 7,374/(1–0.10) = 8,193.

#### 2.1.2 Selection of samples

**Figure 1:**
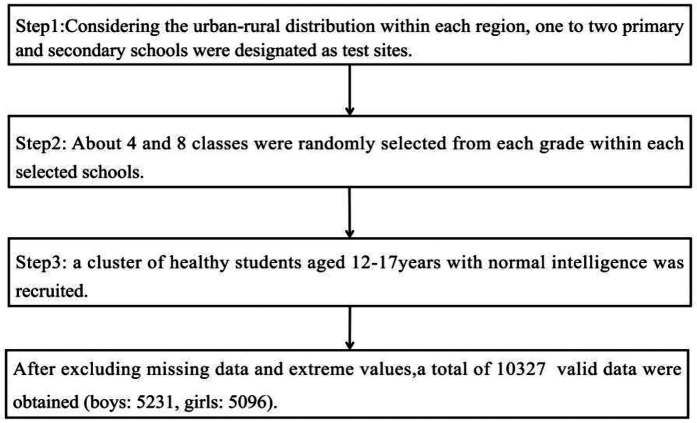
Sample selection flow chart.

From August 2021 to April 2022, 1∼2 junior and senior high schools in each region will be selected as test sites according to urban and rural distribution and geographical distribution in Shanghai, Kunming (Yunnan Province), Changsha (Hunan Province), Suzhou (Anhui Province), and Urumqi (Xinjiang Uygur Autonomous Region). With the natural class serving as the basic sampling unit, a stratified cluster random sampling approach was employed to select between four and eight classes of middle school students from each test site school as participant, resulting in a sample size of approximately 180 male and female individuals across various age groups in each region. With the natural class serving as the basic sampling unit, a stratified cluster random sampling method was utilized to select 4–8 classes of middle school students from each test site school as participants. Approximately 180 male and female participants from various age groups were included in each region. Students in the class who satisfied the research criteria were evaluated through a questionnaire survey. To be eligible for the study, participants were required to meet the following criteria: Informed consent was obtained from all participants. Normal physical function and intelligence, absence of dyslexia, no physical disabilities, and no history of chronic diseases such as hypertension, heart disease, diabetes, or nephritis. The total number of participants was 10,824. After excluding those with incomplete items (*n* = 123) or questionnaires with a missing rate exceeding 5% (*n* = 374), a total of 10,327 valid datasets were retained for analysis (Figure 1), yielding a valid response rate of 95.41%. The final sample included 5,231 boys and 5,096 girls, There was no gender difference in the age of male and female students (Table 1, *P* > 0.05). The investigation carried out by this Research institute was approved by the University Committee on Human Research Protection of East China Normal University with the approval number HR319-2021.

**Table 1:** Distribution of regions, physical activity (PA) and socioeconomic status (SES) of adolescents in China.

Variate	Boys	Girls	Statistical value	*P*
Age ($\bar{x}\;$± SD, years)	14.50 ± 1.71	14.52 ± 1.70	−0.616	0.538
Region [n (%)]				
Shanghai	1,000 (19.1)	1,021 (20.0)	2.319	0.677
Urumqi	1,026 (19.6)	1,004 (19.7)		
Changsha	1,077 (20.6)	1,039 (20.4)		
Kunming	1,040 (19.9)	1,020 (20.0)		
Suzhou	1,088 (20.8)	1,012 (19.9)		
Different Intensity of PA [P50 (P25, P75) min/d]				
LPA	200 (100, 440)	190 (100, 400)	−3.571	<0.001
MPA	225 (100, 450)	177.5 (80, 380)	−8.498	<0.001
VPA	85 (3, 225)	50 (0, 121)	−14.998	<0.001
MVPA	360 (186, 680)	260 (135, 500)	−15.716	<0.001
TPA	675 (404, 1,062)	547.5 (330, 850)	−14.406	<0.001
Different Type of PA [P50 (P25, P75) min/d]				
Entertainment PA	0 (0, 50)	0 (0, 50)	−5.770	<0.001
Transportation PA	308 (150, 545)	270 (140, 500)	−4.438	<0.001
Household PA	5 (0, 45)	20 (0, 54.5)	−8.330	<0.001
Exercise PA	221 (101, 430)	122 (60, 260)	−23.942	<0.001
SES [n(%)]				
Low	1,815 (34.7)	1,767 (34.7)	1.372	0.503
Middle	1,707 (32.6)	1,711 (33.6)		
High	1,709 (32.7)	1,618 (31.8)		

LPA, light-intensity physical activity; MPA, moderate-intensity physical activity; VPA, vigorous-intensity physical activity; MVPA, moderate-to-vigorous-intensity physical activity; TPA, total physical activity.

### 2.2 Methods

#### 2.2.1 The measurement methods for physical activity (PA)

This “Physical Activity Questionnaire for Children and Adolescents Aged 7–18 Years” (21) was used to investigate PA status. The contents of the questionnaire include: PA items, intensity, time and frequency. The questionnaire included 24 PA items. Participants recalled their involvement in various PA over the past week. The intensity of each PA was assessed based on subjective perception (relaxed, slightly tired, very tired) in conjunction with the energy expenditure tables for each activity. Specifically, activities with an energy expenditure of less than 3.0 METs were classified as light intensity, those between 3.0 and 5.9 METs as moderate intensity, and those greater than or equal to 6.0 METs as vigorous intensity. The total duration of light-intensity physical activity (LPA), moderate-intensity physical activity (MPA), vigorous-intensity physical activity (VPA), and total physical activity (TPA) were then calculated. In this study, according to previous studies (22–24), PA was divided into transportation PA (including cycling, walking, climbing stairs, etc.), entertainment PA (including outdoor chasing and playing, shuttlecock kicking, rubber band skipping, etc.), household PA (including sweeping, washing dishes, blackboard cleaning, etc.) and exercise PA (including football, basketball, running, yoga and other sports). Cronbach's *α* coefficients of MVPA and TPA in the survey questionnaire were 0.70 and 0.72, and the retest coefficients were 0.74 and 0.78, respectively. The calibration validity of the questionnaire was tested by accelerometer, and the correlation coefficient was 0.69. All *P* values were < 0.01, which proved that the questionnaire had good reliability and validity. The family economic status was investigated, including parents' occupation, parents' education and family monthly income.

#### 2.2.2 The measurement methods for socioeconomic status (SES) and gender

This study utilized validated questionnaires to assess adolescents' demographic (gender, age, etc) and family characteristics. The SES of participants was assessed using a questionnaire adapted by Fang Xiaoyi (25), which encompasses three primary dimensions: parental educational attainment, parental occupational status, and household income indicators. To ensure measurement reliability, household income was evaluated indirectly through an inventory of household possessions, thereby circumventing potential reporting inaccuracies stemming from adolescents' limited knowledge of their family's actual financial situation. Parental education was categorized into seven distinct levels: (1) no formal education. (2) elementary school. (3) junior high school. (4) senior high school. (5) graduate students and above, and (6) unknown. Parental occupations were classified into 11 categories, including construction workers, civil servants, and educators, among others. The assessment of family facilities included nine commonly owned domestic appliances, with respondents indicating availability through a dichotomous (yes/no) response format.

#### 2.2.3 Quality control

This study utilized standardized and validated questionnaires to conduct the survey. Before initiating the survey, the principal investigator provided standardized training for all investigator. This ensured their familiarity with the questionnaire content and field procedures, thereby minimizing potential errors and enhancing data reliability. During the data collection process, investigators, accompanied by the class teacher or physical education teacher, explained the questionnaire content and provided filling instructions during class meetings or self-study periods. This ensured that any questions raised by students were promptly addressed.

After the questionnaires were completed, the investigators collected them on-site and immediately checked for omissions and corrected any obvious errors. At the end of each day, a review session was conducted to re-examine the questionnaires. Any questionnaires with logical inconsistencies or a missing rate exceeding 5% were excluded. During the data entry phase, double entry was performed using EpiData 3.1. On the same day of data entry, checks for abnormal values were conducted, and any discrepancies were promptly verified against the original questionnaires.

#### 2.2.4 Methods of mathematical statistics

SES calculation method (17): Firstly, score the educational background of parents according to the years of education, which is 5 points for primary school and below, 9 points for junior middle school, 12 points for senior high school, and 15 points for graduate students and above. Parents' occupational status is scored according to the standard of International Standard Occupational Economic Status Index (ISEI) (26). According to the family facilities, one item of family facilities was scored 1, and none was scored 0. Secondly, the parent with the highest years of education and the highest occupational classification score was selected as the representative of the parent's education and occupation. Thirdly, handling missing values for the SES variable: For participants with only one missing value, regression analysis was conducted using the other two variables, and the missing value was imputed using the calculated value from the regression equation. Finally, principal component analysis is then conducted on three variables: parental occupation, parental education, and family monthly income. The specific calculation formula is as follows: SES = (*β*1*Z_parental education_ +*β*2*Z_parental occupation_+*β*3*Z_family monthly income_)/*ε*_ƒ_. *β* is the factor load, *ε*_ƒ_ is the characteristic root. SES scores were divided into three strata—high, middle and low—employing the trichotomy method.

First, the Kolmogorov–Smirnov test was employed to assess the normality of the data. Given that the PA time data did not follow a normal distribution, the Kruskal–Wallis H test, conducted using SPSS version 26.0, was utilized to compare PA times among adolescents across different groups. Following the event, multiple comparisons were conducted using the Mann–Whitney U test. For the two-factor nonparametric analysis, the ScheirerRayHare function from the rcompanion package in R software was utilized. Given that SPSSAU facilitates convenient operations and can automate the generation of dummy variables and interaction terms during the analysis of moderating effects, it was chosen to conduct the moderating effect analysis of gender. The moderator variable (gender) is a categorical variable, it requires dummy variable coding, and the independent variables (SES) were processed to mean centering. First, Model 1 examines the effect of the independent variable (SES) on the dependent variable (PA). Second, Model 2 extends Model 1 by incorporating the moderator variable (gender). Third, Model 3 further extends Model 2 by including the interaction term (SES  ×  Gender) between the independent variable and the moderator variable, thereby examining whether the influence of SES on PA varied between boys and girls.

## 3 Results

### 3.1 The distribution of regions, PA and SES of adolescents in China

Table 1 shows the distribution of regions, PA and SES of adolescents in China. There were no significant differences in age, regional distribution and SES between boys and female students (*P* > 0.05). In terms of different intensity PA, LPA, MPA, VPA, MVPA and TPA time in boys were 200 (100, 440) min, 225 (100, 450) min, 85 (3, 225) min, 360(186, 680) min, 675 (404, 1,062) min, respectively. For girls, the time was 190 (100, 400) min, 177.5 (80, 380) min, 50 (0, 121) min, 260 (135, 500) min, 547.5 (330, 850) min, and the difference between genders was statistically significant (*P* < 0.001 for all). In terms of different types of PA, the daily PA time of traffic, entertainment, housework and exercise for boys was 0 (0, 50) min, 308 (150, 545) min, 5 (0, 45) min and 221 (101, 430) min, respectively. The PA times for girls were 0 (0, 50) min, 270 (140, 500) min, 20 (0, 54.5) min and 122 (60, 260) min, respectively. Boys' PA time in entertainment and housework was lower than that of girls. The PA time of traffic class and exercise class was higher than that of girls (*Z* values were −5.77, −4.438, −8.33, −23.942, all *P* values < 0.001).

### 3.2 Comparison of PA duration at varying intensities among adolescents across different SES groups

Among boys in different SES groups, there were statistically significant differences in PA time of all intensities (all *P* values < 0.05), and the low SES group had the lowest PA time of all intensities. In girls, the difference in PA time among various SES groups was not statistically significant (all *P* values > 0.05); however, the low SES group exhibited a consistently lower level of PA. In general, there were statistically significant differences in PA time of various intensities among all SES groups (all *P* values < 0.01), and the low SES group showed the lowest PA time (see Table 2).

**Table 2:** Comparison of Different Intensity of PA in Adolescents in Different SES Groups [P50 (P25, P75) min/d].

Gender	Variate	LPA	MPA	VPA	MVPA	TPA
Boys	Low SES^a^	195.0 (100.0, 420.0)^c^	210.0 (92.0, 420.0)^c^	75.0 (0.0, 200.0)^b^^,^^c^	339.0 (170.0, 630.0)^b^^,^^c^	645.0 (377.0, 999.0)^b^^,^^c^
	Middle SES^b^	200.0 (100.0, 455.0)	236.0 (110.0, 460.0)	90.0 (10.0, 240.0)^a^	380.0 (200.0, 700.0)^a^	685.0 (420.0, 1,110.0)^a^
	High SES^c^	220.0 (100.0, 460.0)^a^	233.0 (94.5.0, 470.0)^a^	90.0 (10.0, 240.0)^a^	370.0 (192.0, 700.0)^a^	704.0 (416.0, 1,090.0)^a^
	*H*	8.345	8.358	21.293	19.323	20.249
	*P*	0.015	0.015	0.000	0.000	0.000
Girls	Low SES	180.0 (100.0, 390.0)	177.0 (80.0, 380.0)	45.0 (0, 120.0)	258.0 (130.0, 495.0)	530.0 (320.0, 849.0)
	Middle SES	190.0 (100.0, 410.0)	180.0 (80.0, 379.0)	56.0 (0, 125.0)	270.0 (140.0, 500.0)	550.0 (336.0, 851.0)
	High SES	200.0 (100.0, 420.0)	175.0 (80.0, 388.3.0)	60.0 (0, 120.0)	260.0 (135.0, 502.0)	558.0 (330.8, 855.0)
	*H*	1.801	0.390	1.473	1.233	3.068
	*P*	0.406	0.823	0.479	0.540	0.216
Total	Low SES	190 (100,400)^c^	190 (80,400)	60 (0,160)^b^^,^^c^	290 (150,570)^b^^,^^c^	580 (345,925)^b^^,^^c^
	Middle SES	200 (100,423.5)	205 (90,420)	65 (0,180)^a^	320 (160,600)^a^	618 (375,971)^a^
	High SES	200 (100,440)^a^	200 (85,420)	70 (0,180)^a^	310 (155,590)^a^	627 (375,974)^a^
	*H*	9.033	6.013	16.704	13.787	19.033
	*P*	0.011	0.049	0.000	0.001	0.000

P50(P25, P75): 50th percentile (25th percentile, 75th percentile).

SES, socioeconomic status. LPA, light-intensity physical activity; MPA, moderate-intensity physical activity; VPA, vigorous-intensity physical activity; MVPA, moderate-to-vigorous-intensity Physical Activity; TPA: total physical activity.

^a^ Compared with Low SES.

^b^ Compared with Middle SES.

^c^ Compared with High SES.

### 3.3 Comparison of different types of PA time in adolescents in different SES groups

As illustrated in Table 3, for boys, the PA time related to transportation (280 min) and exercise (200 min) in the low SES group was significantly lower than that of other groups, with statistically significant differences (*P* < 0.05). Among girls, the low SES group spent less time in traffic PA (250 min) than the high SES group, and the housework PA (15 min) time was higher than the high SES group, and the differences among the above groups were statistically significant (all *P*-values < 0.05). Overall, the low SES group exhibited the shortest durations for both transportation PA (260 min) and exercise PA (155 min), while household PA was the longest (15 min). These differences were statistically significant (all *P*-values < 0.05).

**Table 3:** Comparison of Different types of PA in Adolescents in Different SES Groups [P50(P25, P75) min/d].

Gender	Different SES groups	Statistical value	Entertainment PA	Transportation PA	Household PA	Exercise PA
Boys	Low SES		0.0 (0, 50.0)	280.0 (140.0, 520.0)^b^^,^^c^	10.0 (0, 50.0)	200.0 (100.0, 390.0)^b^^,^^c^
	Middle SES		0.0 (0, 40.0)	310.0 (160.0, 560.0)^a^	1.0 (0, 40.0)	240.0 (110.0, 450.0)^a^
	High SES		0.0 (0, 43.5)	340.0 (152.5, 560.0)^a^	0.0 (0, 42.0)	240.0 (106.0, 470.0)^a^
		*H*	4.348	15.510	5.409	25.589
		*P*	0.114	0.000	0.067	0.000
Girls	Low SES		2.0 (0, 54.0)	250.0 (140.0, 470.0)^c^	20.0 (0, 60.0)^c^	120.0 (60.0, 250.0)
	Middle SES		0.0 (0, 45.0)	266.0 (140.0, 510.0)	20.0 (0, 60.0)	128.0 (60.0, 261.0)
	High SES		0.0 (0, 42.8)	300.0 (150.0, 510.0)^a^	15.0 (0, 50.0)^a^	125.0 (55.8, 265.3)
		*H*	5.578	8.688	12.516	1.329
		*P*	0.061	0.013	0.002	0.514
Total	Low SES		0 (0, 50)^c^	260 (140, 500)^b^^,^^c^	15 (0, 50)^b^^,^^c^	155 (71, 315)^b^^,^^c^
	Middle SES		0 (0, 45)	300 (145, 532.8)^a^	10 (0, 50)^a^	178 (78.8, 355)^a^
	High SES		0 (0, 42)^a^	310 (150, 530)^a^	10 (0, 50)^a^	175 (75, 360)^a^
		*H*	8.378	21.682	14.487	16.972
		*P*	0.015	0.000	0.001	0.000

P50(P25, P75): 50th percentile (25th percentile, 75th percentile).

SES, socioeconomic status; PA, physical activity.

^a^ Compared with Low SES group, *P* < 0.05.

^b^ Compared with Middle SES group, *P* < 0.05.

^c^ Compared with High SES group, *P* < 0.05.

### 3.4 The interaction between gender and SES significantly influenced the MVPA index

**Figure 2:**
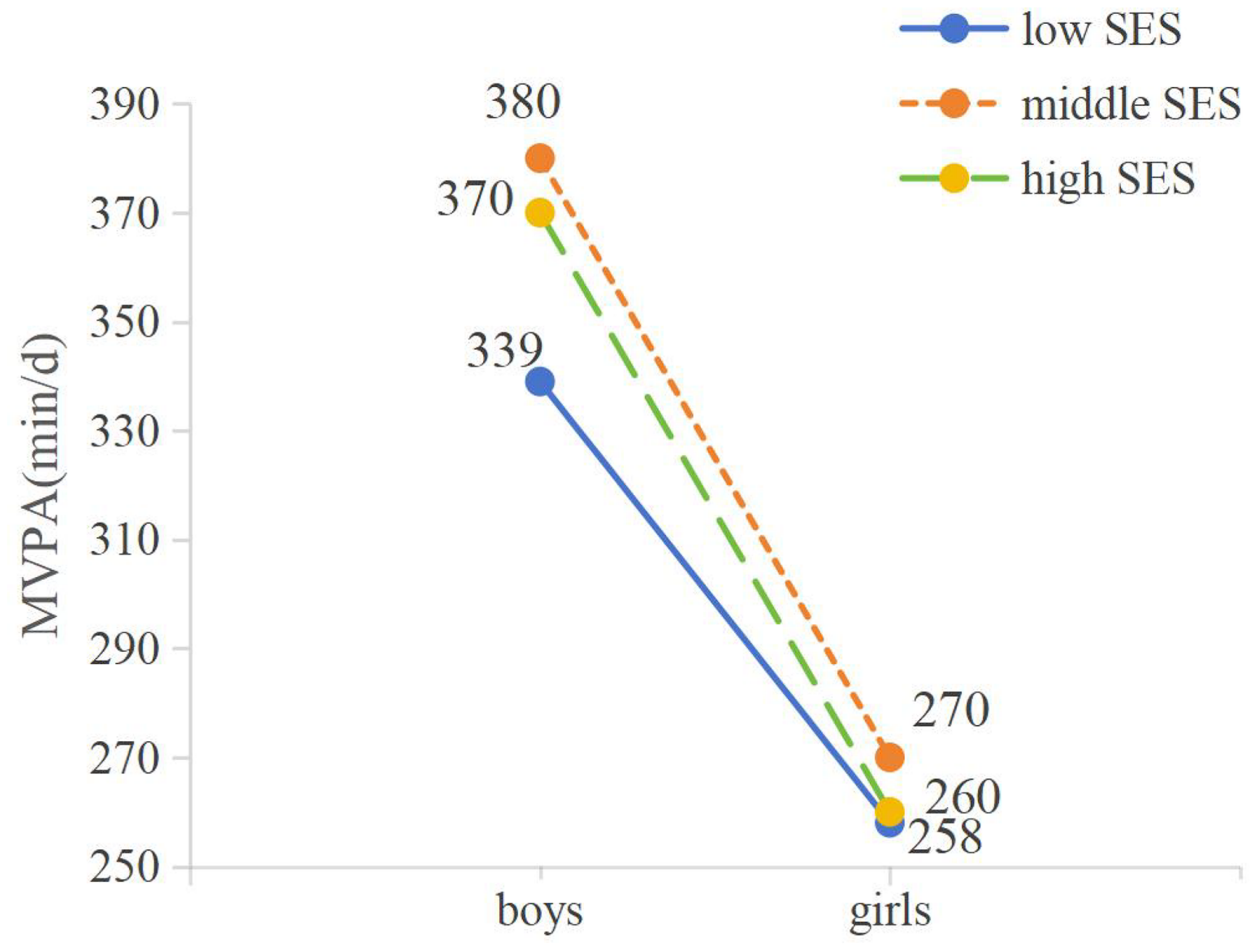
Levels of Moderate to Vigorous Physical Activity (MVPA) among adolescents of different genders and family socioeconomic status (SES).

As illustrated in Table 4, the results of the Scheirer-Ray-Hare test reveal statistically significant differences in MVPA metrics across different genders (H = 247.377, *P* < 0.001), and there were statistical differences in MVPA between different SES groups (H = 14.175, *P* < 0.001). The interaction between gender and SES significantly influenced the MVPA metrics (H = 6.495, *P* *<* 0.05). Figure 2 shows the Levels of MVPA among adolescents of different genders and SES. MVPA time of boys in different SES groups was higher than that of girls, and MVPA time of adolescents in middle SES group was higher than that of high and low SES groups.

**Table 4:** Interaction of gender and SES on MVPA.

Variate	Df	Sum Sq	*H*	*P*
Gender	1	2.20 × 10^+9^	247.377	<0.001
SES	2	1.26 × 10^+8^	14.175	<0.001
Gender*SES	2	5.77 × 10^+7^	6.495	0.039

SES, socioeconomic status.

**Table 5:** Analysis of moderating effects of gender and SES on MVPA.

Variate	model 1					model 2					model 3				
	Bs	SE	*t*	*P*	β	B	SE	*t*	*P*	β	B	SE	*t*	*P*	β
Constant	473.921	15.000	31.596	0.000	-	539.891	15.448	34.948	0.000	-	540.633	15.45	34.992	0.000	-
SES	16.046	4.935	3.252	0.001	0.034	16.953	4.88	3.474	0.001	0.036	26.024	6.574	3.959	0.000	0.055
Gender						−141.461	9.254	−15.287	0.000	−0.149	−141.445	9.252	−15.287	0.000	−0.149
SES Gender											−19.141	9.296	−2.059	0.040	−0.028
R^2^	0.002					0.024					0.025				
F value	F (3,10317) = 7.296, *p* = 0.000					F (4,10316) = 64.017, *p* = 0.000					F (5,10315) = 52.078, *p* = 0.000				

Adjust for region and age. SES, socioeconomic status.

### 3.5 The moderating influence of gender on the relationship between SES and MVPA

In view of the above analysis, the interaction between gender and SES is established, and gender may be a moderating variable of SES and MVPA, so the moderating effect analysis is conducted. After controlling for relevant variables in Model 3, the interaction term coefficient between gender and SES was statistically significant (*B* = −19.141, *t* = −2.059, *P* *<* 0.05), suggesting a moderating effect of gender on the relationship between SES and MVPA. Specifically, when the gender was female, the association between SES and MVPA was attenuated (see Table 5).

## 4 Discussion

This study demonstrated that boys exhibited significantly higher levels of PA and TPA across all intensity levels compared to girls, particularly in transportation PA and exercise PA. Regarding the MVPA and TPA duration among adolescents, individuals from low SES groups exhibited a trend of lower engagement compared to those from middle and high SES groups. Specifically, the low SES group exhibited lower levels of transportation PA and exercise PA compared to the other two groups. Importantly, gender moderates the relationship between SES and MVPA, with different SES levels having a more pronounced effect on PA in boys than in girls.

Previous research results are consistent (27, 28). MVPA in Chinese boys is higher than that in girls, and it is mainly reflected in transportation PA and exercise PA. The reasons may be attributed to the distinct physiological and psychological developmental characteristics of boys and girls, as well as their differing social roles. There are gender differences in behavior attitude and MVPA behavior intention of male and female students. Male students exhibit a tendency to actively engage in sports, whereas female students generally display a less positive attitude toward physical activities (29). However, in the growth and development stage, a reasonable level of PA is conducive to the growth and development of adolescents. Therefore, particular attention should be given to the phenomenon of inadequate PA levels among girls. Efforts should focus on guiding and encouraging girls to actively engage in diverse forms of PA, with special emphasis on transportation and exercise PA.

While previous research (13, 30, 31) has consistently shown that boys tend to engage in higher levels of MVPA compared to girls, and that SES is positively correlated with PA levels, this study offers several novel insights.

First, contrary to earlier findings from a 2018 survey of Chinese-adolescents (24), which suggested that low SES groups engaged in higher levels of transportation PA, our study found that adolescents from high SES families exhibited higher levels of both transportation and exercise PA. This shift may reflect changing societal attitudes toward health and PA, particularly among higher-educated parents who increasingly prioritize their children's participation in structured exercise and active transportation. There is a positive correlation between parents' educational background and children's health literacy (32). Parental education may play a major role in the self-regulation of adolescents' active participation in exercise. Parents with higher education have a deeper understanding of health cognition and health behaviors, so they have a stronger awareness of participating in exercise, and often require or accompany their children to do it together. Parents actively participate in and support their children's exercise. Their children are more likely to convert their intention to participate in sports exercise into actual behavior (33), thereby playing a significant role in promoting the children's engagement in physical exercise (34). Trost (23) et al. 's and Jenum (13) et al.' s studies both believe that people with high SES are more active in PA than those with low SES, and SES is positively correlated with PA. Adolescents from high SES families have a higher level of PA, often because the higher the level of SES in the family, the more complete the sports facilities near the residence and the school, so they can have more opportunities to participate in sports (35). Furthermore, adolescents from low SES families may have reduced access to parks and recreational areas in their residential neighborhoods, or live in a community with less walking environment or other fitness facilities (36). Consequently, they have fewer opportunities to engage in physical exercise and sports skills (37). These factors likely contribute to the lower levels of PA observed among adolescents from low SES families compared to those from middle and high SES families.

Second, Our findings reveal that the relationship between SES and PA is gender-specific, with SES exerting a stronger influence on boys' PA levels than on girls'. This gender moderating effect has not been extensively explored in prior studies.

The influence of SES on PA levels is more pronounced among boys, likely due to the combined effects of multiple factors, including societal gender roles, family resource allocation, school and community environments, cultural pressures, and psychological and behavioral differences. Such as: Boys in low SES communities exhibit significantly lower participation in PA during breaks compared to their counterparts in high SES communities, whereas girls' participation is less influenced by SES (35). In low SES communities, the scarcity of sports facilities disproportionately impacts boys, who are more reliant on external facilities to engage in PA (37), thereby resulting in a more pronounced reduction in their PA levels (36). In contrast, girls tend to engage more in activities that do not require specialized facilities, which may partially explain why the association between SES and PA levels is more pronounced among boys.

This findings suggest that tailored approaches are needed to address the unique barriers faced by girls and boys. For girls, it is essential to focus on cultivating their interest in PA, particularly in transportation and exercise PA. Schools and communities could offer gender-sensitive programs that cater to girls' preferences and interests, such as Cheerleading, yoga, or team sports, to increase their engagement in PA. Considering the substantial influence of SES on boys' PA, targeted interventions should focus on supporting boys from lower SES. These interventions could include providing access to affordable sports facilities, organizing community-based PA programs, and promoting active transportation options in boys with low SES. Efforts should focus on providing structured opportunities for PA, such as after-school sports clubs or subsidized access to recreational facilities.

Additionally, since parental education background and health awareness play a important role in shaping adolescents' PA behaviors, parental involvement should be a key component of future interventions. For instance, health education initiatives could be implemented to enhance parental awareness regarding the advantages of sustained PA, thereby motivating them to actively engage in or facilitate their children's exercise regimens.

The current research, while offering valuable insights, is not without its limitations. It should be acknowledged that, despite the substantial sample size, the reliability and stability of self-report measures are inferior to those of objective measurement methods. In future research, it is recommended to incorporate a combination of large sample sizes, objective testing methods, and self-report measures to enhance the comprehensiveness and robustness of the investigation. Second, this study utilized a cross-sectional survey design, which precludes the identification of causal relationships. Future research should consider prospective long-term longitudinal follow-up studies to better analyze the various factors influencing PA.

Future research should continue to explore the mechanisms underlying these relationships, particularly the reasons why boys' PA levels are more sensitive to SES. By doing so, we can develop more effective strategies to promote PA and reduce health disparities among adolescents.

## 5 Conclusion

In conclusion, adolescents from low SES backgrounds exhibited the lowest levels of MVPA and TPA, which were mainly manifested in transportation PA and exercise PA. Gender moderates the relationship between SES and MVPA, with different SES levels having a more pronounced effect on PA in boys than in girls.

## Data Availability

The raw data supporting the conclusions of this article will be made available by the authors, without undue reservation.
